# Glia Regulate the Development, Function, and Plasticity of the Visual System From Retina to Cortex

**DOI:** 10.3389/fncir.2022.826664

**Published:** 2022-02-01

**Authors:** Nicholas Benfey, David Foubert, Edward S. Ruthazer

**Affiliations:** Department of Neurology and Neurosurgery, Montreal Neurological Institute-Hospital, McGill University, Montreal, QC, Canada

**Keywords:** astrocyte, Müller glia, microglia, visual system, ocular dominance plasticity, neuron–glia interactions, retina, visual cortex

## Abstract

Visual experience is mediated through a relay of finely-tuned neural circuits extending from the retina, to retinorecipient nuclei in the midbrain and thalamus, to the cortex which work together to translate light information entering our eyes into a complex and dynamic spatio-temporal representation of the world. While the experience-dependent developmental refinement and mature function of neurons in each major stage of the vertebrate visual system have been extensively characterized, the contributions of the glial cells populating each region are comparatively understudied despite important findings demonstrating that they mediate crucial processes related to the development, function, and plasticity of the system. In this article we review the mechanisms for neuron-glia communication throughout the vertebrate visual system, as well as functional roles attributed to astrocytes and microglia in visual system development and processing. We will also discuss important aspects of glial function that remain unclear, integrating the knowns and unknowns about glia in the visual system to advance new hypotheses to guide future experimental work.

## General Introduction

**FIGURE 1:**
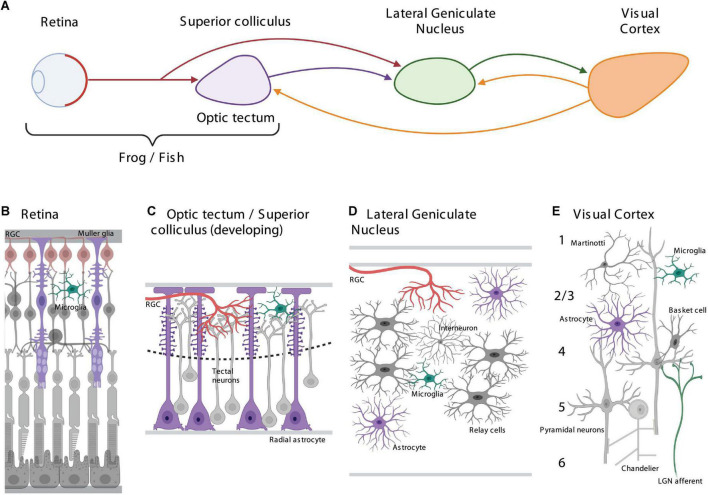
Schematic representations of the vertebrate visual system and the astrocytes and microglia populating each area. **(A)** Overview of the vertebrate visual system showing the connectivity between major visual areas, in fish and frogs, the retina and optic tectum; and in mammals, the retina, superior colliculus, lateral geniculate nucleus, and the primary visual cortex. **(B)** Cellular organization of the retina showing Müller glia and microglia. **(C)** Cellular organization of the optic tectum/developing superior colliculus showing radial astrocytes and microglia. **(D)** Cellular organization of the lateral geniculate nucleus showing astrocytes and microglia. **(E)** Cellular organization of the primary visual cortex showing astrocytes and microglia.

The vertebrate visual system has long been used as a highly tractable system in which to study how sensory experience influences the development, function, and plasticity of neurons and neural circuits in the brain. Over the past several decades, the development of novel tools and techniques has begun to allow for the precise monitoring and targeted manipulation of glial cells throughout the central nervous system and has led to a series of rapid advances in our understanding of the significant ways in which glia actively contribute to brain development and function, including in multiple areas of the visual system. As a result, glial cells, such as astrocytes and microglia, are now becoming widely accepted as critical regulators of a multitude of behaviorally relevant neural processes. Throughout all areas of the developing and adult brain, including the visual system, glial cells are found in close apposition to neurons where they contact synapses, influencing their formation, maturation, and function (Figure 1). In this review article, we will discuss what is known (and unknown) about the roles of Müller glia, astrocytes, and microglial cells in shaping the development, function, and plasticity of the vertebrate visual system, focusing on mechanisms mediating neuron-glia communication and their functional roles in each stage of the system from the retina to the primary visual cortex.

## Introduction of the Vertebrate Visual System

In the back of each eye, specialized photoreceptive retinal tissue senses and responds to distinct features of light energy relaying detailed information about wavelength, intensity, spatial location, and motion to the brain (Figure 1A). The structure and function of the retina is highly conserved between vertebrate species with the outer layer mediating the majority of photoreception through activation of rod and cone photoreceptor cells and the inner layer predominantly processing and relaying that visual information to the brain through selective activation of bipolar and retinal ganglion cells (RGCs) (Masland, 2012). A parallel light-sensing pathway responsible for entrainment of circadian rhythms and other non-image-forming functions is mediated by melanopsin phototransduction in a class of intrinsically photosensitive RGCs (Berson et al., 2002; Hattar et al., 2002; Lazzerini Ospri et al., 2017). Müller glia are the dominant glial cell in the retina having an elongated radial morphology that spans both the inner and outer layers, extending out numerous filopodial processes into synaptic layers which form contacts with retinal synapses (Wang et al., 2017)(Figure 1B).

The retina connects to the brain through the RGC axons which bundle together within the optic nerve (Figure 1A). In fish and frogs which develop externally and whose behaviors rely on vision throughout early development, the vast majority of RGC axons cross the optic chiasm and project contralaterally, innervating a constellation of midbrain pretectal nuclei and most prominently the optic tectum – a retinotopically organized structure homologous to the mammalian superior colliculus – where they form synapses upon tectal neurons (Baier and Wullimann, 2021). The primary function of the optic tectum or superior colliculus involves integrating sensory information and correctly orienting an animal’s behavioral responses in space (Ito and Feldheim, 2018; Isa et al., 2021). Similar to in the retina, the predominant glial cells in the optic tectum of fish and frogs, radial astrocytes (also referred to as radial glia), have an elongated radial morphology which spans from the ventricular to the pial surfaces of the brain, extending highly dynamic filopodia that contact synapses in the neuropil layer (Tremblay et al., 2009) (Figure 1C).

In the mammalian brain, RGC axons innervate specific laminae within both the superior colliculus and the lateral geniculate nuclei (LGN) of the thalamus, along with the intergeniculate leaflet and pulvinar nucleus, collectively referred to as the visual thalamus (Hooks and Chen, 2020) (Figure 1A). The visual thalamus transmits visual information to the primary visual cortex, and also receives input from several other brain areas including both the superior colliculus and layer 6 of the primary visual cortex. While the LGN is known to communicate eye-specific information related to color and spatiotemporal features of visual information, a complete understanding of the function of the LGN is still being elucidated, with experiments suggesting it plays more nuanced roles than just acting as a simple linear filter (Kerschensteiner and Guido, 2017). The LGN is populated by both astrocytes and microglia, with astrocytes exhibiting mature morphological characteristics by the time of eye opening (Somaiya et al., 2021) (Figure 1D).

Thalamic relay neurons in the LGN receiving input from the retina project their axons as a large fiber tract, called the optic radiation, into the primary visual cortex (V1), as well as some higher cortical areas depending on the species, where they largely innervate layer 4 with a smaller subset innervating layers 2/3 and 6 while preserving retinotopic spatial organization (Espinosa and Stryker, 2012) (Figure 1A). The topographic organization of the primary visual cortex in large mammals, including most carnivores and primates, is highly structured across its surface. Within the primary visual cortex neurons participate in hypercolumns consisting of multiplexed maps of spatial frequency, orientation, and ocular dominance where groups of neurons exhibit eye preference and selective responsiveness to diverse stimulus features, for each region of the visual field (Espinosa and Stryker, 2012). The primary visual cortex is largely believed to act as a preliminary feature detector used to create a saliency map of objects in the visual field (Zhang et al., 2012). The layers of the primary visual cortex are extensively populated by both astrocytes and microglia (Parnavelas et al., 1983) (Figure 1E).

As we will discuss in detail in the following sections, in the eye, Müller glia have been found to both respond to and modulate spontaneous retinal waves which are known to be instructive in the refinement of visual connections throughout the developing brain, and along with microglia, to contribute to the proper transduction of signals through the retina as well as to direct its repair following injury. In retinorecipient areas such as the optic tectum (superior colliculus) and the LGN, astrocytes and microglia respond to sensory stimulation and influence synapse maturation and the segregation of retinal inputs through activity-dependent mechanisms. In the primary visual cortex astrocytes and microglia are responsive to both visually-evoked neuronal activity and the release of neuromodulators. They regulate the excitability of cortical neurons and gate the duration of critical periods for plasticity. With defined mechanisms for glial functions at each stage of the visual system starting to be revealed, the potential for synthesizing a preliminary model integrating how all of these different types of glia contribute to visual experience across the visual system is now an emerging possibility. We will begin this review by highlighting what is currently known about the activity-dependent functions of glial cells in each major segment of the visual system and then follow with a discussion of the major outstanding questions remaining in the field and experiments that would help to bridge these important gaps before finishing with a predictive summary model for how they contribute to the development and function of the visual system.

## The Adult Retina – Müller Glia

Müller glia constitute one of the most highly studied glial cell types given the relative ease of access to intact retinal tissue for experimental manipulation. It has long been appreciated that they are responsive to visually evoked activity and mediate diverse roles in retinal development, function, and both degeneration and regeneration.

Early work demonstrated that in the retina the glutamate transporter GLAST (EAAT1) is expressed exclusively by Müller glia and required for proper signal transduction between photoreceptors and bipolar cells, indicating that they actively contribute to the processing of visual information in the circuit (Harada et al., 1998). Shortly thereafter, experiments in isolated retinal preparations demonstrated that Müller glia respond to mechanical stimulation with increases in calcium which propagated between retinal Müller glia by means of the release of extracellular ATP, hinting that glia may also be an active signaling partner capable of influencing retinal function through the release of such compounds during periods of stimulation (Newman, 2001). Later, and again in isolated retinal preparations, Müller glia were found to exhibit spontaneous calcium transients while under constant illumination that could be increased by either presenting a flickering light stimulus or through antidromic stimulation of RGCs (Newman, 2005). These calcium transients could also be blocked by the purinergic antagonist suramin or the voltage-gated sodium channel blocker tetrodotoxin (TTX), demonstrating that their calcium activity is subject to regulation by sensory-driven neuronal activity and hinting that it may occur physiologically *in vivo*. Investigation into the mechanism by which light activates Müller glia found that ectonuclease-mediated breakdown of ATP to adenosine increases their calcium responses, which could be prevented by apyrase, an enzyme that limits availability of ATP for adenosine generation. Conversely, blocking mGluRs, NMDARs, or mAChRs, many of the main mediators of visually-driven activity in retinal neurons, had no effect on calcium activity in Müller glia (Newman, 2005). Consistent with neuronal activity driving the calcium increases in Müller glia, transients were observed to start in their fine processes in the synaptic layers before traveling to their endfeet at the inner surface of the retina (Newman, 2005). Using novel bioluminescent assays that allowed for the calcium activity of Müller glia to be observed under conditions of darkness, investigators found that Müller glia exhibited temporally-coordinated patterns of spontaneous calcium activity that repeated over time in small networks of cells and that this could be blocked by TTX suggesting that spontaneous neural activity drives the activation of Müller glia in structured ways (Agulhon et al., 2007).

The first demonstration that calcium transients occur in Müller glia under physiological conditions *in vivo* also observed that they were mediated through the propagation of extracellular ATP, as treatment with apyrase reduced their occurrence by 95%, confirming that the isolated retina was an informative preparation for investigating the mechanisms mediating the activity of Müller glia (Kurth-Nelson et al., 2009). A luciferin assay was then used to quantitatively monitor the release of ATP during spontaneous calcium waves in the rat retina and found that ATP release occurred during the increases in calcium activity in Müller glia and that the amount and frequency of its release increased across development (Kurth-Nelson et al., 2009). Importantly, adenosine has been shown to hyperpolarize RGCs suggesting that the activity-dependent release of ATP from retinal glia may modulate retinal function under physiological conditions (Clark et al., 2009).

Igniting much interest at the time, the spontaneous calcium waves in Müller glia were observed to co-occur with changes in the diameter of retinal arterioles suggesting the activity of Müller glia may control neurovascular coupling in the retina (Kurth-Nelson et al., 2009). There has been much discussion surrounding the role of astrocytic calcium in mediating blood vessel dilation throughout various areas of the brain (Bazargani and Attwell, 2016), and later experiments in the retina helped to address aspects of this controversy by showing that retinal capillaries, but not arterioles, which were adjacent to Müller glial endfeet, dilate following visually evoked calcium responses and that knockout of IP3R2 (the major mediator of calcium release from internal stores in most glia) prevents light evoked dilation of capillaries but not arterioles (Biesecker et al., 2016).

While a mechanistic understanding of the functions mediated by calcium transients in glia remains an active focus in neuroscience research generally, particularly the activity- and calcium-dependent release of gliotransmitters from various glia throughout the brain, functional manipulations in Müller glial cells have contributed some important insights. The targeted expression of botulinum toxin, a protease that cleaves and inactivates the SNARE proteins necessary for normal calcium-mediated vesicular release, in Müller glia demonstrated that calcium causes the vesicular release of glutamate, but not ATP from Müller glia (Slezak et al., 2012). Although no differences in retinal structure or visual processing were observed when vesicular release of glutamate from Müller glia was prevented, the authors did suggest that their lack of ability to target all Müller glia with botulinum toxin in their experimental preparations may have prevented any such alterations (Slezak et al., 2012), a suggestion consistent with recent observations that only sparse activation of radial astrocytes in the hindbrain of zebrafish is required to fully mediate behavioral effects (Mu et al., 2019).

Structurally, Müller glia span across the entire width of the retina and there have been several interesting observations suggesting that since most cells and structures in the retina cause light scattering, Müller glia may act as biological optic fibers that can help to guide light to the photoreceptor layer in the retina of adult guinea pigs (Franze et al., 2007). Furthermore, additional experiments showed that Müller glia are capable of acting as wavelength-specific guides for light in the isolated retina, channeling red-green spectrum light to cone photoreceptors while allowing blue-purple spectrum light to bleed into areas populated by rod photoreceptors (Labin et al., 2014). Whether this plays a physiological role in the intact retina *in vivo* has yet to be demonstrated.

In the zebrafish retina Müller glia have become an important subject of study following observations that they can act as late-stage progenitor cells that can functionally repopulate damaged retina in adult fish (Bernardos et al., 2007), something that is not normally possible in the adult mammalian retina despite a high degree of similarity in structure and function (Karl et al., 2008). This work has started to identify promising avenues for restoring function in the damaged mammalian retina as the transcription factor Ascl1 has been shown to be upregulated in Müller glia in the retina of zebrafish following injury and to mediate the regeneration of retinal tissue; while importantly, no such expression of Ascl1 occurs in mammals following retinal damage (Karl et al., 2008). Encouragingly, it has been shown that when Ascl1 is overexpressed in Müller glia in mice, along with an inhibitor of histone deacetylase, retinal regeneration is possible following injury suggesting a potential avenue to induce retinal regeneration in mammals (Karl et al., 2008).

The interactions between microglia, the resident immune cell in the central nervous system, and Müller glia also appear to mediate important roles in retinal development and repair as the presence of microglia is required for the regenerative potential of Müller glia in the adult zebrafish retina (Conedera et al., 2019). Additionally, endocannabinoids, known to modulate glia throughout the CNS, have also been suggested to play a role in mediating the proliferative function of Müller glia as pharmacological activation of Müller glia endocannabinoid receptors promotes the formation of progenitor cells without having an observable impact on microglial responses in the damaged retina (Campbell et al., 2021).

## The Developing Retina – Müller Glia

Much of what is known about the function of Müller glia comes from experiments done in the adult retina; however, more recent work has started to explore their function throughout development as well, offering important mechanistic insights that complement work done in the mature system.

In the developing mouse retina, Müller glia have been shown to exhibit dynamic calcium waves in response to both cholinergic and glutamatergic retinal waves mediated by mAChRs or AMPARs respectively (Rosa et al., 2015). Interestingly, mAChRs were not observed to regulate the calcium activity of Müller glia in the adult retina (Newman, 2005), suggesting that the mechanisms underlying the responsiveness of Müller glia to neural activity are plastic throughout development. As the retina continues to mature, Müller glia increase their expression of excitatory amino acid transporters leading to a continual decrease in the spillover of synaptic glutamate which results in a corresponding decrease in the activation of Müller glial AMPARs (Rosa et al., 2015). Müller glia in the retina of larval zebrafish have also been shown to exhibit spontaneous electrical and calcium waves mediated by the activation of glial AMPARs, beginning in their fine processes in the synaptic layer before spreading vertically to their cell body and endfoot and then horizontally between other Müller glial cells – likely through the release of ATP – while Müller glial excitatory amino acid transporters were observed to tightly regulate the occurrence and propagation of neuronal retinal waves, suggesting that the activity of Müller glia may play a permissive role in the generation of the spontaneous retinal waves that are known to direct the topographic refinement of RGC axons into retinal recipient areas of the brain (Zhang et al., 2019).

Structurally Müller glia are complex cells with numerous fine filopodial processes extending into the surrounding neural retina which are highly motile throughout development (Tworig et al., 2021 and see review in this Research Topic). Whether calcium activity in glial cells regulates the structural motility of their processes had remained an open question until recently. Here it was shown that calcium activity is compartmentalized in Müller glial stalks (the elongated radial portion of the cell spanning the width of the retina) and processes, with M1 mAChRs mediating calcium events in Müller glial stalks but not in processes (Tworig et al., 2021). Curiously, acute manipulation of retinal wave activity had no effect on lateral process motility in Müller glia, and chronic manipulation had no impact on the distribution, complexity, or length of glial lateral processes, together suggesting a functional decoupling between structural motility and calcium signaling as wave-associated compartmentalized calcium activity was not required for, nor did it regulate Müller glial process motility (Tworig et al., 2021).

## The Retina – Microglia

In the embryonic mouse retina, microglia have been found to primarily associate with newly born neurons and the depletion of retinal microglia leads to an increase in the density of RGCs without altering the proliferation of progenitor cells or the birth of new RGCs suggesting that microglia actively phagocytose RGCs as a normal part of development in the retina (Anderson et al., 2019). These effects of microglia on prenatal development of neurons have been shown to be mediated through complement proteins, known to mediate neuronal phagocytosis and pruning by microglia in other brain areas (Anderson et al., 2019; Lim and Ruthazer, 2021). In the developing retina, knockout of the cytokine receptor CX3CR1 in microglia has been shown to lead to retinal dysfunction shortly after eye opening and the eventual loss of cone type photoreceptors expressing its signaling partner fractalkine during postnatal development (Jobling et al., 2018). CX3CR1 knockout produced detectable changes in the morphology of retinal microglia as well as the structural elongation of photoreceptors (Jobling et al., 2018).

In the adult retina of mice, acute depletion of microglia does not appear to impact the structural organization or architecture of the retina nor does it appear to impact the survival of retinal neurons (Wang et al., 2016). However, prolonged depletion of retinal microglia was observed to lead to the degeneration of photoreceptor synapses and causes a progressive loss of proper light induced visual responses in the retina (Wang et al., 2016). Additional work has demonstrated that the Fractalkine-CX3CR1 signaling pathway regulates the repopulation and reestablishment of functional roles of newly born microglia in the retina following depletion (Zhang et al., 2018). Microglia and cytokine signals have also been shown to be required for the formation of Müller glia-derived progenitor cells in response to injury in the mature retina as differentiation does not occur in animals where microglia have been ablated (Fischer et al., 2014).

## The Lateral Geniculate Nucleus – Astrocytes

Compared to the retina, the functional roles of glia in the mammalian brain regions that receive retinal input, such as the superior colliculus and LGN, remain less well characterized, especially in intact animals likely due to their relative inaccessibility compared with other regions of the visual system. During development, RGC axons from both eyes topographically innervate the LGN occupying overlapping territories and synapsing promiscuously with thalamic neurons before patterned neural activity instructs their proper segregation and refinement into tightly organized laminae with the correct postsynaptic partners (Guido, 2008). In the developing LGN of mice, astrocytes are positioned and appear morphologically mature, having ensheathed retinogeniculate synapses and being capable of glutamate uptake, before eye-opening (Somaiya et al., 2021).

Several studies have now demonstrated that astrocytes orchestrate synaptogenesis, axonal segregation, and synaptic refinement in this circuit. An early indication that astrocytes actively promote the generation of functional synapses came from *in vitro* work where purified RGCs were cultured with and without astrocytes. This study found that astrocytes mediate increases in the overall number of synapses per RGC, as well as enhanced vesicular release and synaptic efficacy (Ullian et al., 2001). While these observations were purely made *in vitro*, the authors did mention that the appearance of SV2-positive synaptic puncta occurs at the same time as the appearance of S100β positive astrocytes suggesting their observations are likely to be relevant *in vivo*. Subsequent work identified thrombospondins as the astrocyte-secreted signal responsible for their promotion of synaptogenesis in the CNS of rodents (Christopherson et al., 2005). In addition to *in vitro* analyses of synaptogenic effects of thromobspondins on cultured RGCs, they further confirmed the expression of thrombospondins throughout the postnatal visual system and demonstrated that animals deficient in thrombospondins have deficits in synapse formation *in vivo*.

One of the first studies to directly implicate astrocytes in axonal segregation and refinement *in vivo* came from work characterizing the role of the complement protein C1q in synapse elimination in the developing LGN which found that disruptions in either C1q or its signaling partner C3 lead to the excessive innervation of thalamic neurons by RGC axons in rodents (Stevens et al., 2007). Hunting for the source of C1q in the circuit, they discovered that immature astrocytes were necessary for inducing the upregulation of all C1q subunits in RGC axons, ultimately targeting them for elimination.

Further evidence consolidating a role for astrocytes in mediating the activity-dependent segregation of retinal inputs in the mouse LGN came a few years later when it was demonstrated that not only do astrocytes actively tag retinal inputs for elimination, but they also directly phagocytose synaptic connections tagged for elimination as well (Chung et al., 2013). The researchers found that the proteins MEGF10 and MERTK were localized to astrocytes and these signaling pathways mediate phagocytic engulfment and pruning of synapses by astrocytes during development. Similar to disruptions in C1q or C3, knockout of MEGF10 or MERTK in astrocytes was also shown to lead to impaired segregation and the accumulation of abnormal weak inputs onto thalamic neurons in the LGN. Interestingly, this pruning of synapses by astrocytes was demonstrated to be neural activity-dependent with astrocytes continuing to engulf synapses in the adult brain suggesting synaptic pruning by astrocytes is not restricted to critical periods of development.

The activity of retinal inputs in the developing LGN of mice has also recently been shown to regulate the recruitment of interneurons into the circuit through the activation of astrocytes (Su et al., 2020). Mechanistically, the activity of RGCs was found to drive the expression of FGF15 in astrocytes with FGF15 ultimately being shown to be the necessary signal mediating the recruitment of interneurons into the thalamus, a process critical to proper function of the LGN.

## The Optic Tectum – Radial Astrocytes

The amphibian retinotectal circuit is a well-characterized model for studying how visual experience regulates neurodevelopment and plasticity in the intact brain of living vertebrates. In the brain of the African claw-toed frog *Xenopus laevis*, RGC axons innervate the optic tectum, an area analogous to the mammalian superior colliculus, forming synaptic connections with the dendrites of tectal neurons. Radial astrocytes are the principal resident glial cell, which form columnar zones tiling the optic tectum where they act as a hybrid cell type mediating the roles of both radial glial progenitor cells and astrocytes (Tremblay et al., 2009).

In *X. laevis*, the regulation of the proliferative roles of radial astrocytes by visual activity has been studied. It was demonstrated that the generation of new radial astrocytes expressing the proliferative marker Musashi1 decreases as the optic tectum matures, and that 48 h of visual deprivation leads to an increase in the number of Musashi1 positive radial astrocytes, enhancing proliferation (Sharma and Cline, 2010). Visual stimulation also increases their differentiation into new tectal neurons ultimately showing that visual experience tightly regulates the development of the optic tectum through the activation of radial astrocytes. The knockdown and overexpression of Musashi1 in radial astrocytes have shown that it is both necessary and sufficient for proliferation of radial astrocytes in the developing amphibian visual system; however, the mechanism through which these cells detect and process visual information was not investigated in this study (Sharma and Cline, 2010).

Radial astrocytes have been shown to extend hundreds of fine filopodia into both neuropil and cell body layers of the frog optic tectum that contact synapses between RGC axons and tectal neuron dendrites (Tremblay et al., 2009). Radial astrocyte filopodia are highly motile and exhibit continuous structural remodeling over short timescales. Radial astrocytes also exhibit spontaneous calcium transients and both filopodial motility and calcium fluctuations can be increased through visual stimulation and reduced by blocking neuronal NMDARs or nitric oxide synthase. Additional studies have demonstrated that neuronal NMDAR activation mediated effects on radial astrocyte filopodial motility through signaling by the CyclicGMP-Dependent Protein Kinase 1 (PKG-1) and that the motility of radial astrocyte filopodia decreases throughout development (Sild et al., 2016). Manipulating the PKG-1 signaling pathway through expression of dominant-negative PKG-1 in radial astrocytes led to reductions in the frequency of mEPSCs in neighboring neurons, indicative of a failure to undergo normal synaptic maturation (Sild et al., 2016). Additionally, when radial astrocyte filopodia were eliminated through the expression of a constitutively active RhoA this led to reductions in the frequency and amplitude of mEPSCs in neighboring neurons while the expression of a dominant negative Rac1 that reduced the motility of radial astrocyte filopodia resulted in reduced density of synapses onto neighboring neurons demonstrating that activity-dependent filopodial motility in radial astrocytes is an important contributor to neuronal maturation and function in the optic tectum (Sild et al., 2016).

The roles of gliotransmitter release in shaping neuronal development and function are hotly debated in the neuroscience community. The roles of the NMDAR co-agonist D-serine have been studied in the *X. laevis* optic tectum where it has been found to be present within both neurons and radial astrocytes and to modulate NMDAR-mediated retinotectal synaptic signaling (Van Horn et al., 2017). In the rodent hippocampus, D-serine, thought to undergo vesicular release from astrocytes in response to calcium transients, has been shown to promote synaptic long-term potentiation through its enhancement of NMDAR-mediated currents (Henneberger et al., 2010). Rearing tadpoles in exogenous D-serine promoted the developmental maturation of retinotectal synapses, a process involving the trafficking of AMPA receptors to the synapse through mechanisms akin to LTP (Van Horn et al., 2017). This finding further indicated that under normal physiological conditions D-serine levels are not saturating. Furthermore, degradation of D-serine by the addition of exogenous D-amino acid oxidase enzyme prevented normal synapse maturation in the tectum, findings that implicate gliotransmission as playing a role in normal circuit maturation.

In addition, enhancement of NMDAR function further leads to the hyperstabilization of retinotectal axonal arbors in the optic tectum, presumably through the action of a retrograde signal (Van Horn et al., 2017). At the functional level, D-serine increases the size of receptive fields for ON visual responses in tectal neurons, further indicating this putative gliotransmitter is important for the functional maturation of the visual system. Whether and how D-serine is released from radial astrocytes in response to neuronal activity remains an active area of investigation.

Advances in genetically-encoded calcium indicators such as GCaMP6, which can be used for high resolution functional imaging of neurons and glia *in vivo* (Stobart et al., 2018), and new tools for the semi-automated segmentation of cellular regions of interest based on activity have recently allowed for the systematic investigation of the molecular signaling pathways underlying sensory-driven neuron-glia communication during real time in the intact visual system of *Xenopus laevis* tadpoles (Benfey et al., 2021). Using the expression of GCaMP6s throughout both tectal neurons and radial astrocytes, which are morphologically distinct, allowing for clear segmentation of signal, calcium activity in both cell types has been monitored by live imaging (Benfey et al., 2021). Radial astrocytes were shown to exhibit spontaneous calcium transients in the segments of the cell embedded in the tectal neuropil, an area rich in retinotectal synapses. These transients are almost entirely abolished by treatment with the voltage-gated sodium channel blocker TTX, suggesting they are generated in response to neural activity. Radial astrocytes in the optic tectum were further shown to be highly responsive to visual stimulation, exhibiting temporally-correlated increases in calcium activity that lagged behind neuronal activation by several seconds. Visually-evoked calcium responses were observed to be better correlated between neighboring radial astrocytes suggesting their activity patterns reflect the topographical organization of retinal inputs into the tectum during early development. Somewhat unexpectedly, blockade of all glutamate receptors in the optic tectum of young tadpoles abolished visually-evoked responses in tectal neurons but not radial astrocytes, whereas the blockade of either glial excitatory amino acid transporters or sodium calcium exchangers (NCX) abolished visually-evoked responses in radial astrocytes.

## The Lateral Geniculate Nucleus and the Optic Tectum – Microglia

Relatively little is known about the roles of microglia in mediating the development and function of retinal recipient areas such as the geniculate and optic tectum; however, several studies in both mouse, fish and frog have suggested that they are likely to be mediating important effects related to the refinement of synapses in these areas during development.

In the developing LGN of mice, microglial processes have been observed to extend out close to serotonergic axon terminals where they sense and respond to focal serotonin release through 5HT2B receptors (Kolodziejczak et al., 2015). In 5HT2B knockout mice, there are observable alterations in the refinement of ipsilaterally-projecting retinothalamic connections, and markers for microglial activation are also increased; however, given this knockout was not specific to microglia, it remains a possibility these effects are unrelated to their function (Kolodziejczak et al., 2015).

Microglia in the mouse geniculate have also been shown to regulate synapse removal through a non-phagocytic pathway involving fn14 and TWEAK expression in neurons and microglia, respectively (Cheadle et al., 2020). Here neuronally-expressed fn14 was found to promote the formation of bulbus spines on thalamic relay neurons thereby strengthening connections between RGCs and the visual thalamus; however, when microglia expressing TWEAK interacted with fn14 on thalamic neurons, synaptic connections were weakened and ultimately eliminated demonstrating that microglia can mediate the refinement of synaptic connections through mechanisms independent of phagocytosis (Cheadle et al., 2020).

In the *X. laevis* optic tectum, microglia have been observed to surveil the tectal neuropil where they contact and accumulate material from intact RGC axons (Lim and Ruthazer, 2021). Depleting microglia was shown to lead to an increase in the number of branches on RGC axonal arbors, likely through a decrease in pruning, and quite interestingly to an inversion of expected behavioral responses to light and dark looming stimuli suggesting that microglial interactions with RGC axons are critical for the refinement of circuitry responsible for proper execution of important escape behaviors. RGCs in *Xenopus* tadpoles were shown to express a complement inhibitory protein, amphibian regulator of complement activation 3 (aRCA3), a homolog of mammalian CD46. Overexpression of aRCA3 in RGCs reduced the accumulation of axonal material by microglia and increased the size of RGC axonal arbors while over expression of complement C3 at RGC axon synapses led to a decrease in axonal arbor size, demonstrating that microglial interactions with RGC axons are regulated by complement proteins and appear to participate in developmental structural refinement.

## The Primary Visual Cortex – Astrocytes

Consistent with observations from various areas across the cortex, astrocytes in the primary visual cortex (V1) each occupy adjacent territories, tiling the volume of the brain. Whether astrocytes in V1 have overlapping territories with neighboring astrocytes was recently explored in the visual system of ferrets. Here it was observed that despite clear tiling of astrocytes throughout V1, most of the astrocytes have territories that overlap with their neighbors by about 50% suggesting it may be possible for multiple astrocytes to differentially influence a shared subset of neighboring neurons (López-Hidalgo et al., 2016). Interestingly, a specific subset of astrocytes in layers 3 and 4 that receive thalamic input, referred to as kissing astrocytes, were found to have territories that overlap substantially less than those in other layers of V1 demonstrating that astrocyte tiling does not apply uniformly across the cortex and is likely to have important influences on neuronal function (López-Hidalgo et al., 2016). Work by a group using computational models to help understand how the spatial distribution of astrocytes influences the organization of the visual cortex found that when astrocytes were integrated into a model for the formation of orientation preference maps, by simply altering the radius of astrocytes one can alter the radius of lateral excitatory connections ultimately modifying the size and presence of orientation preference maps in the visual cortex (Philips et al., 2017).

It is likely that astrocytes contribute to the formation of functional maps throughout the visual system in multiple ways given that they express important axon guidance molecules such as ephrins and their receptors (Murai and Pasquale, 2011) and form spatially compartmentalized networks through gap junctions (Houades et al., 2008; Roux et al., 2011).

In the developing brain, the density of astrocytes increases in the primary visual cortex until around the time of eye opening, and gap junctions between astrocytes appear to be present before birth (Wang et al., 2021). Early research into the development of astrocytes in the visual system observed that dark-rearing can delay their maturation (Müller, 1990). Subsequently, an extensive characterization of astrocyte development and maturation in the visual system observed that only long periods of dark-rearing (a minimum of 4 weeks) were sufficient to change the membrane properties of astrocytes in the primary visual cortex, specifically through altering the expression of astrocytic potassium channels (Wang et al., 2021). Binocular deprivation was found to increase gap junctional coupling between astrocytes, and similarly to dark-rearing, it only occurred if it was maintained for at least 4 weeks.

Another recent study performed in depth characterization of how the transcriptional regulation of astrocytes is influenced by neuronal activity across development of the visual cortex. They observed that significant changes in the transcriptome of astrocytes correlated with changes in the expression of genes associated with the formation of synapses, and that the expression of genes in astrocytes that regulate synapse formation are modulated by both thalamic neuronal activity and calcium activity in the astrocytes (Farhy-Tselnicker et al., 2021). Consistent with the observations discussed above, they also observed that astrocytes exhibit transcriptional heterogeneity in the visual cortex based on spatial location, and, that consistent with the larger literature on astrocytes, both neuronal and astrocytic activity alters the transcriptome of astrocytes in ways that suggest effects beyond just those related to synapse formation.

Astrocytes also contribute to the development of synaptic connections in the cortex. Astrocytes have been shown to help mediate the linkage of presynaptic neurexins and postsynaptic neuroligins between developing thalamocortical synapses in V1 (Singh et al., 2016). Astrocytes accomplished this by releasing the protein Hevin, which along with the protein SPARC has previously been shown to influence the organization of pre- and postsynaptic connections between the retina and the superior colliculus (Kucukdereli et al., 2011). Astrocyte-secreted Hevin directly induced the formation of thalamocortical synapses by acting as a structural bridge linking together neurexin 1α with neuroligin 1. Additionally it was demonstrated that the recruitment of neuroligin 1 and NMDARs to excitatory synapses *in vivo* also relies on astrocyte released Hevin, and that, as will be relevant to the section of this review on astrocytes and ocular dominance plasticity, Hevin released from astrocytes is necessary for ocular dominance plasticity in the developing brain. More recently, astrocyte-secreted chordin-like 1 has also been shown to drive the maturation of synapses in the visual cortex by inducing the switch from calcium-permeable AMPARs to calcium-impermeable AMPARs (Blanco-Suarez et al., 2018).

## How Astrocytes in the Primary Visual Cortex Respond to Neuronal Activity

Some of the first direct evidence that astrocytes respond to sensory-evoked neuronal activity *in vivo* came from observations in the primary visual cortex of juvenile ferrets (Schummers et al., 2008). Here it was shown that astrocytes reliably respond to visual stimulation with increases in internal calcium levels which were mediated by glial excitatory amino acid transporters and which lagged behind neuronal responses by several seconds – observations which are highly similar to those observed in radial astrocytes in the developing optic tectum mediated through excitatory amino acid transporters and sodium-calcium exchangers (Benfey et al., 2021) suggesting the possibility of a conserved mechanism mediating both visually-evoked responses. Surprisingly, the visually-evoked calcium responses in astrocytes were found to exhibit highly-refined stimulus-feature selectivity and receptive field properties which were previously only attributed to neurons (Schummers et al., 2008). In fact, it was found that similar to neurons, astrocyte orientation selectivity was finely mapped across the visual cortex and that astrocytes have even sharper tuning for spatial-frequency and orientation than the surrounding neurons.

Several years later astrocytes in the primary visual cortex of mice were also shown to respond to visual stimulation; however, visually-evoked responses in these astrocytes were very weak when animals were immobile and it was only during forced locomotion that visually-evoked responses became robust (Paukert et al., 2014). In fact, forced locomotion was shown to induce coordinated wide-scale activation of calcium transients in astrocytes throughout the mouse brain, with norepinephrine being found to mediate this effect, suggesting that locomotion, (or norepinephrine) gates the responsiveness of astrocytes to sensory-evoked stimulation in the mouse visual cortex (Paukert et al., 2014).

This dual influence of locomotion and visual stimulation on the activity of astrocytes in the primary visual cortex has been replicated several times now with additional insights into the mechanism having been characterized. Astrocytes in the visual cortex of mice were again shown to respond directly to visual stimulation and also to exhibit high amplitude alpha-1 adrenergic receptor mediated global calcium events throughout the cortex in response to norepinephrine released from the locus coeruleus which tended to mask the lower amplitude visual stimulation induced responses (Sonoda et al., 2018; Slezak et al., 2019). Similar to the kinetics observed in the responses of astrocytes in the primary visual cortex of ferrets, visually-evoked events in astrocytes were delayed by approximately 5 s relative to neuronal responses suggesting the likelihood of a conserved mechanism underlying the responses in both species (Sonoda et al., 2018). Interestingly, they observed distinct patterns of calcium activity in astrocytes in response to locomotion and visual stimulation and that chemical ablation of norepinephrine releasing neurons abolished locomotor but not visually-evoked responses (Slezak et al., 2019). The mechanism mediating visually-evoked events in astrocytes was not explored in this context, however. It is worth noting that even though astrocytes in the primary visual cortex of mice do respond to visual stimulation, unlike those found in ferrets, they do not appear to exhibit any clear stimulus-feature selectivity (Asada et al., 2015), likely due to a comparative lack of functional organization in the visual cortex of mice (López-Hidalgo et al., 2017).

Astrocytes in the visual cortex also exhibit extensive spontaneous activity, particularly within small microdomains of their fine processes even in the absence of locomotion or visual stimulation. The signals mediating spontaneous microdomain activity in astrocytes have been hotly debated (Bazargani and Attwell, 2016). Researchers have found that different neurotransmitters activate distinct microdomains within cortical astrocytes (Agarwal et al., 2017). However, spontaneous calcium events in these microdomains continued to occur when neurotransmitter release was prevented or when the release of calcium from internal stores was blocked pharmacologically or largely abolished in IP3R2 knockout mice (Agarwal et al., 2017). Interestingly, spontaneous events in microdomains of the fine processes of astrocytes were found to colocalize with mitochondria, and ultimately, the mitochondrial membrane permeability transition pore was found to be responsible for generating spontaneous microdomain calcium events that occur in the absence of neurotransmitter release. Thus, astrocytes may exert effects on neural circuits as a consequence of intrinsic metabolic activity (Agarwal et al., 2017), in response to neuromodulatory release during states of heightened arousal (Paukert et al., 2014; Slezak et al., 2019), or as a direct response to sensory-evoked neuronal activity (Schummers et al., 2008; Paukert et al., 2014; Sonoda et al., 2018; Slezak et al., 2019).

## Action of Astrocytes on Visual Cortical Response Properties

Several studies have examined how selectively activating astrocytes in the primary visual cortex using optogenetic tools regulates the firing and response properties of cortical neurons and shapes visual processing. Optogenetic activation of astrocytes in mouse V1 was found to enhance synaptic transmission in layers 2/3 through the activation of presynaptic mGluR1, likely as a consequence of astrocytic glutamate release (Perea et al., 2014). Photoactivation of astrocytes led to increases in mEPSC frequency in both PV+ and SOM+ inhibitory neurons and modulated their spontaneous activity along with that of excitatory neurons in primary visual cortex *in vivo*. Activation of astrocytes was found to alter both excitatory and inhibitory drive onto neurons throughout V1. Consequently, visual responses and orientation selectivity of cortical neurons were changed by astrocyte activation, with increasing baseline responsiveness leading to a corresponding decrease in orientation selectivity. Interestingly not all neurons responded identically, with PV+ interneurons uniformly increasing baseline visual responses, while SOM+ interneurons showed bidirectional changes. All of these effects of astrocyte activation could be prevented by systemic blockade of mGluR1a, consistent with a central role for astrocytic glutamate release.

Other studies investigated how the activity of astrocytes influences the firing activity of neurons throughout the layers of the primary visual cortex of mice. Consistent with the findings reported above, the firing activity of neurons in layers 2/3 of V1 was increased following 4 weeks of dark rearing and, surprisingly, this was found to be linked to increased gap junction coupling between astrocytes (Wang et al., 2021). Another group, using optogenetic activation of astrocytes in mice, observed increases in calcium responses, depolarization, and spiking in layer 5 pyramidal neurons accompanied by potentiation of inputs onto their apical dendrites in layer 1 (Ryczko et al., 2021). Interestingly these effects were found to persist in the presence of blockers for glutamate, GABA and ATP receptors suggesting they were mediated by a non-classical mechanism. Ultimately it was demonstrated that astrocytes mediate these effects by reducing extracellular calcium around neurons through the activity-dependent release of the calcium binding protein S100β, highlighting the remarkable diversity of mechanisms for neuron-glia communication.

## Roles of Astrocytes in Regulating Plasticity in the Primary Visual Cortex

The possibility that astrocytes might regulate developmental plasticity in the mammalian visual cortex first attained prominence with the observation that ocular dominance plasticity could be restored after the normal critical period had closed by grafting immature astrocytes cultured from the visual cortex of kittens into the visual cortex of adult cats (Müller and Best, 1989). In line with this observation, dark-rearing, which is known to delay critical period closure in the visual cortex, was demonstrated to lower the levels of astrocytic markers such as GFAP and S-100 compared to normal light-rearing, suggesting both that the maturation of astrocytes is linked to visual experience and that there is an association between the presence of immature astrocytes and the potential for plasticity in the visual cortex (Müller, 1990).

More recently there has been a significant advance in our understanding of how astrocytes regulate ocular dominance plasticity by helping to gate the opening and closure of the critical period. Researchers revisited the early observation that the transplantation of immature astrocytes into adult visual cortex, this time in the mouse, can restore ocular dominance plasticity (Ribot et al., 2021). They first observed that the intrinsic membrane properties of astrocytes mature by postnatal week 3, just before the onset of the critical period for ocular dominance plasticity in mice, and additionally, that immature astrocytes undergo a significant shift in transcriptional regulation at this time moving away from the expression of genes regulating cell division and toward the expression of genes regulating cellular communication. Transcripts for proteins involved in the formation of gap junctions were some of the most highly differentially expressed genes, with CX30 exhibiting the highest representation. They showed that CX30 levels increase throughout development and peak at the time of closure of the critical period of ocular dominance plasticity. Four days of exposure to darkness reduced CX30 expression in V1 and increased plasticity, while knockdown of CX30 in astrocytes in V1 delayed the closure of the critical period significantly from P28 to P50. Additionally, grafting immature astrocytes lacking CX30 reopened the critical period in adult mice. When taken together, these observations demonstrate a critical effect of astrocytic expression of the gap junction protein CX30 in gating plasticity in the visual cortex. The researchers took these observations a step further by investigating the role of CX30 in the maturation of PV+ interneurons in V1, which are known to be important regulators of ocular dominance plasticity. Knockdown of CX30 in astrocytes led to a decreased ratio of inhibition to excitation in the cortex and significant alterations in perineuronal nets, a known marker for the maturation of PV+ interneurons and plasticity. They concluded the study by demonstrating that astrocytic CX30 regulates the formation of perineuronal nets through a pathway involving RhoA and release of the extracellular matrix metalloprotease MMP9. Taken together the results of this study demonstrate a critical role for astrocytes in gating cortical plasticity by regulating the maturation of PV+ interneurons consistent with the larger literature on ocular dominance plasticity.

As discussed above, glutamate transporters have been shown to mediate responsiveness of astrocytes to visual stimulation in the primary visual cortex of ferrets (Schummers et al., 2008); as such, understanding how astrocytic glutamate transporters influence the development of the visual system is of particular interest. GLT-1 (EAAT2) is the dominant glutamate transporter expressed by astrocytes in the visual cortex of mice, and visual experience has been shown to increase GLT-1 expression levels in V1 astrocytes throughout development (Sipe et al., 2021). In GLT-1 heterozygous (HET) mice, astrocytic levels of GLT-1 are reduced by approximately 50% in the visual cortex. In these mice the normal developmental matching of orientation preferences of the responses of the two eyes in V1 cells is disrupted. Layer 2/3 neurons show increased spine density, suggestive of a reduced amount of developmental synaptic pruning. Furthermore, ocular dominance plasticity is significantly altered by this change in the expression of astrocytic GLT-1, with responses to the non-deprived eye failing to increase, and paradoxically decreasing over time, highlighting the important role played by astrocytic glutamate uptake for normal plasticity in the developing visual cortex.

The neuromodulator acetylcholine has also long been known to play a critical role in cortical plasticity in the visual system (Bear and Singer, 1986). Responses to a visual stimulus can be strongly potentiated in excitatory neurons in the visual cortex of adult mice by pairing the release of acetylcholine by nucleus basalis stimulation with the visual stimulus (Chen et al., 2012). It was determined that this potentiation of visual responses is dependent upon the activation of muscarinic acetylcholine receptors on astrocytes in the visual cortex. Importantly, knockout of IP3R2 in these cells abolished this phenomenon, indicative of a role for astrocytic calcium in mediating the effects of cholinergic-mediated cortical potentiation.

Astrocytes have also been implicated in injury-induced plasticity of the adult visual system which can be induced by monocular enucleation (ME), the surgical removal of one eye, in which, following a period of quiescence in the cortical territory serving the lost eye, gradual functional reorganization restores responsiveness to other inputs (Hennes et al., 2020). ME has been shown to lead to a rapid increase in the density of astrocytes in primary visual cortex, suggesting they are likely to be contributing to the ability of the cortex to reorganize following ME. This was confirmed by metabolic silencing of astrocytes with fluoroacetate immediately following ME and observing that the functional recovery of neurons in the visual cortex was significantly impaired as a result.

## Microglia in the Primary Visual Cortex

In the visual cortex astrocytes are not the only glial cell type that has been shown to respond to visual activity and influence circuit development and function. Microglia are also appreciated to contribute to the function of the visual system in important ways (for a dedicated review on microglia and the development of the visual system see Dixon et al., 2021). Here we have included some of the relevant contributions of microglia to the development and function of the visual cortex.

Several studies have investigated the behavior of microglia in response to normal or restricted visual experience. During normal visual experience microglia in the visual cortex of juvenile mice extend numerous processes that are associated with synapses at small spines and which are surrounded by extracellular space (Tremblay et al., 2010). Following light deprivation and eventual reexposure, microglia can be observed at the ultrastructural level to exhibit alterations in the extracellular space around synapses, increases in phagocytic markers, and enhanced contact with synaptic clefts. Additionally, light deprivation caused microglia to exhibit less motility and shift their contacts to larger spines, while light exposure reversed these effects. Microglial morphology and process motility have also been shown to change quickly following monocular deprivation and lead to alterations in their interaction with synapses (Sipe et al., 2016). The morphology, territories, and behavior of microglia in the visual cortex continue to undergo changes as animals age suggesting potential roles across the life span of animals (Tremblay et al., 2012).

Several mechanisms associated with microglial function have been explored in the visual cortex of mice. The purinergic receptor P2Y12 is exclusively expressed by microglia in the visual cortex and has been shown to be required for normal ocular dominance plasticity (Sipe et al., 2016). P2Y12 knockout reduces ramification of microglia but does not affect baseline motility of their processes. It prevents the increase in microglial ramification observed during monocular deprivation which is normally associated with enhanced plasticity. The CX3CR1 receptor too has been shown to play important roles in microglial effects on circuit formation and function in areas of the brain such as the retina, but whether it is a global regulator of microglial function is still being investigated. It was observed that knockout of CX3CR1 in microglia does not impair the segregation of retinal inputs in the thalamus and that visual acuity is normal in these animals. Additionally, the activity-dependent potentiation of synaptic connections is normal in CX3CR1 knockout mice as well as the effects of monocular deprivation on ocular dominance plasticity and thalamocortical and cortical synapse density, together suggesting that CX3CR1 does not play an important role in microglial function in the visual cortex relevant to plasticity (Schecter et al., 2017), observations which have been replicated (Lowery et al., 2017).

Of particular interest given the considerable impact of norepinephrine release on astrocyte function in the visual cortex, norepinephrine has also been shown to act as a potent regulator of microglial function in the mouse visual cortex. The first indication came with the observation that microglial surveillance was significantly increased during anesthesia, and that the alpha-2 adrenergic receptor agonist dexmedetomidine, which reduces norepinephrine release, increased microglial surveillance similarly to anesthesia (Stowell et al., 2019). Further investigation into the signaling pathways regulating these effects found that beta-2 adrenergic receptor activation decreases the motility of microglia, and that blockade of beta-2 adrenergic receptors in awake mice reproduces the effects of anesthesia on increasing microglial surveillance. Importantly, the activation of beta-2 receptors was shown to impair the ability of microglia to respond to injury, and chronic activation of microglial beta-2 receptors impaired ocular dominance plasticity in adolescent mice.

Finally, as discussed above, astrocytes have been found to help close the critical period for plasticity in the visual cortex through the regulation of perineuronal nets (Ribot et al., 2021). Interestingly, the depletion of microglia in the adult mouse brain also leads to an increase in the density of perineuronal nets and produces many of the same effects observed when activating astrocytes in the visual cortex, such as increases the amplitude of EPSCs in excitatory pyramidal neurons, increases in local excitatory connections onto excitatory pyramidal neurons, and increases the number of inputs from PV+ interneurons onto excitatory neurons in V1 which would all be consistent with reduced plasticity in the circuit (Liu et al., 2021). Together this strongly suggests, as has recently been observed in other parts of the cortex (Badimon et al., 2020), that astrocytes and microglia may signal to each other to mutually alter the functional properties of neurons in the visual cortex.

## Future Perspectives

Although many outstanding questions remain regarding the functional roles of glia throughout the visual system, particularly in deep brain structures such as the thalamus, a clear picture is starting to emerge from the available literature in which glia, through sensing and responding to visually-evoked neuronal activity, directly influence the connectivity, maturation, function, and plasticity of neuronal circuits in each major station of the visual system. As we continue to refine our mechanistic understanding of how they shape these processes, important conceptual gaps in our knowledge are becoming apparent and point toward meaningful avenues for future research. In the following section we will highlight what we believe to be the most salient open-ended questions yet to be addressed in the field related to the extended functions of glia across the nuclei of the visual system.

Given that information is relayed through the visual system in stages, and that glial activity influences neuronal function locally within each nucleus of the visual system, the possibility that glial activity at one site might influence neuronal activity in another distal location in the visual system is worth seriously investigating. With new techniques allowing for precise targeting and (in)activation of glial cells in intact behaving animals becoming more prevalent, it should now be possible to study potential long-range influences that glia may exert. For addressing these issues, there are particular advantages to the use of highly optically accessible model systems such as *X. laevis* and *Danio rerio* where the entire visual system can be imaged at once.

Given that Müller glia have been shown to regulate the excitability of retinal neurons and modulate the retinal waves known to instruct the topographic and eye-specific refinement of axonal inputs in retinorecipient areas, an important open question is whether the modulation of the signaling pathways that act on Müller glia (e.g., excitatory amino acid transporters, AMPARs, mAChRs, and calcium) can alter the structural and functional connectivity of retinofugal and thalamocortical projections. In theory it should be possible to precisely manipulate the signaling pathways in Müller glia and investigate whether there are alterations in the retinotopic innervation and structural refinement of axons innervating the tectum (superior colliculus) or the LGN and whether these manipulations significantly influence the functional properties of these circuits by impacting such things as neurotransmitter release from RGCs, receptive field properties of postsynaptic neurons, and the maturation of retinotectal or retinogeniculate synapses during critical periods of development. More nuanced investigation into whether controlling the functional activation of Müller glia can alter the information content of visual signals being relayed from the retina to the tectum (superior colliculus) and thalamus, and from the thalamus to the cortex would also be illuminating. Behavioral testing could also contribute novel information about the extent to which glial function in one region of the nervous system can exert an influence on sensory processing in developing and mature animals. Whether there is a difference between any such Müller glia-mediated effects in animals such as fish and frogs, where visual experience is instructive throughout their development, and mammals where instead, spontaneous retinal activity before eye-opening is instructive throughout early development, would be equally interesting to explore (Pratt et al., 2016). The roles of microglia in each of these experimental manipulations should also be characterized given their influence on photoreceptor function and Müller glial maturation. Logically, such investigations should also be extended to glia in retinorecipient areas as well, characterizing their influence on structural and functional connectivity between the tectum (superior colliculus), the LGN, the visual cortex, and perhaps even back onto the retina.

Glia in retinorecipient areas are dramatically understudied in comparison to glia in the retina and visual cortex despite critical activity-dependent roles having been identified for glial function in areas such as the tectum (superior colliculus) and the LGN. As such, an in-depth characterization of the different signaling pathways mediating glial activity in these areas is warranted to better understand what information they are integrating and how they are sensing and responding to visual experience across multiple temporal and spatial scales. Focus should be directed toward characterizing which ion exchangers, neurotransmitter and neuromodulator receptors and transporters are expressed by these glial cells, what heterogeneity exists in their expression across development, and how this correlates with changes in neural circuitry over the same developmental epochs. Particular attention should be paid to signaling pathways that are regulated by visual activity *in vivo*, as our recent work in the *Xenopus* tectum suggests that canonical signaling pathways regulating glial calcium activity identified *in vitro*, while still functional *in vivo*, may not actually contribute in significant ways to the integration of sensory-evoked neural activity (Benfey et al., 2021).

Neuromodulators such as norepinephrine and acetylcholine, long implicated in regulating the plasticity of the visual system, are emerging as powerful regulators of glial activity throughout the vertebrate brain in some cases directly mediating their effects on plasticity through the activation of astrocytes and microglia (Van Horn et al., 2021). As such, a full characterization of which neuromodulators signal through glia to influence neuronal function in each part of the visual system may be critically important for a comprehensive understanding of how plasticity is mediated throughout the visual system as a representative example of sensory processing in general. Whether different neuromodulators, such as acetylcholine or norepinephrine, have distinct influences on glial activity and function within and across different segments of the visual system is currently unexplored and may be particularly instructive. During the development of the visual system, astrocytes have been shown to influence the excitability, connectivity, and maturation of both excitatory and inhibitory neurons, but whether this influence also extends to the neuromodulatory axons innervating these areas of the visual system and whether glia may regulate the release of neuromodulators remains an open question. As both neuromodulators and astrocyte gap junctions have been implicated in gating critical periods of plasticity in the visual system, how neuromodulators influence the expression and maturation of astrocyte gap junctions, and whether this relationship is present throughout the visual system, may provide additional insight into the mechanisms by which both influence plasticity in these circuits.

Throughout different regions of the visual system, as has been observed more generally throughout the central nervous system, neural activity elicits responses in glia through a variety of different types of signaling mechanisms at different stages of development which, despite largely converging at the level of intracellular calcium dynamics over distinct spatiotemporal patterns of release, suggests the existence of extensive heterogeneity between glia both within and across different segments of the visual system. This warrants careful attention and extensive characterization in order to better understand the precise ways in which glia respond to and influence neuronal activity in distinct locations and at distinct times throughout development. Of particular interest is whether all glial cells in each region of the visual system are responsive to both visually-evoked neuronal activity and the release of neuromodulators or whether distinct subpopulations of glia exist, each with unique stimulus sensitives and functional influences on neurons. Whether the activation of glia by sensory-evoked neural activity alone, the release of neuromodulators alone, or through co-activation by both stimuli simultaneously, results in different functional outcomes such as distinct influences on proliferation and neuronal differentiation, as well as the release of specific gliotransmitters over different spatio-temporal scales, has important implications for understanding how glia exert their influences over circuit development, maturation, function, and plasticity.

Given that astrocytes and microglia in the visual system are responsive to neuromodulatory signals known to regulate complex processes such as learning, memory, and attention, whether glia in different segments of the visual system play a role in mediating higher order processes such as perceptual learning, metaplasticity, and the encoding of salient visual information remains an interesting avenue for future experimental work. More generally, the information content contained within patterned glial activity within networks of the visual system remains largely unexplored despite early experiments showing that astrocytes in the primary visual cortex of ferrets exhibit highly refined stimulus-feature selectivity and receptive field properties. Astrocytes may represent complex information in ways complementary and possibly unique from those used by neurons and across different spatiotemporal scales as well. Whether glia in other areas of the visual system also exhibit refined stimulus-feature selectivity remains to be investigated further.

In each region of the visual system, the activity of astrocytes and microglia are both modulated by largely overlapping signaling pathways often mediating complementary influences on neuronal circuit development and function. Exploring to what extent, and through what means, astrocytes and microglia both independently and mutually influence the developing and mature visual system to regulate neural circuit formation and function will be important to carefully consider in all future investigations.

## Conclusion

**FIGURE 2:**
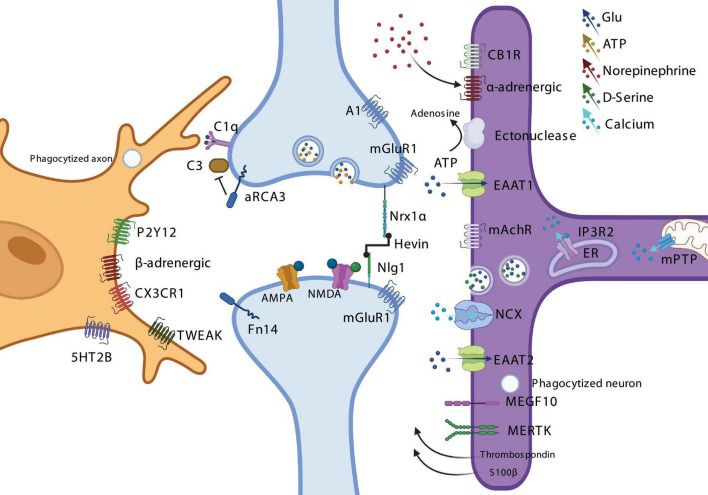
Overview of the signaling mechanisms implicated in neuron-glia communication and interactions throughout the vertebrate visual system. Representative microglial cell (Left, orange), pre- and postsynaptic terminals (Middle, blue), and Müller glial cell/radial astrocyte/astrocyte (Right, purple) expressing a variety of receptors, transporters, channels, and signaling proteins relevant to their functions across the visual system.

Despite the extensive heterogeneity in the morphologies of glia (Figures 1B–E) and the host of molecular signaling pathways mediating neuron-glia communication across brain regions and development (Figure 2), there appear to be important evolutionarily conserved functions mediated by glia throughout the visual system which are likely to be of general relevance to other sensory systems in the vertebrate brain. Throughout the visual system, astrocytes and microglia both sense and respond to neural activity through parallel mechanisms, integrating local circuit activity and the release of neuromodulators such as norepinephrine, acetylcholine, serotonin, and ATP/Adenosine in order to instruct the segregation and refinement of afferent axonal projections through both phagocytic and non-phagocytic means, as well as the genesis, maturation, maintenance, and plasticity of synaptic connections through a series of physical and chemical interactions. Taken together this suggests that astrocytes form a complex network of dynamic integrators which actively influence the development and function of the nervous system by linking together bottom-up sensory-evoked neuronal activity, largely through the reuptake of neurotransmitters, with top-down context-specific neural activity, through their activation by neuromodulators, at the level of intracellular calcium dynamics.

Having largely mapped out what is known and unknown about how glia influence the development and function of the visual system, we will conclude with some speculation as to how visual experience would be altered without functional glia in each segment of the system. Without functional Müller glia and microglia in the retina the spontaneous waves of activity that instruct the topographic refinement of retinal inputs into our brains during early development may be considerably dysregulated, possibly leading to imprecise spatial maps of our visual world, and ultimately disrupting how we detect and track the motion of objects across space. Additionally, we would also quickly go blind as we age as a result of rapid deterioration of photoreceptors without Müller glia and microglia to maintain their synaptic integrity. Without functional radial astrocytes and microglia in the tectum, we know that synapses will not properly mature, and that receptive fields would be significantly altered, together likely leading to defects in the crucial opto-motor responses necessary to direct our attention as well as detect and orient ourselves toward or away from threats in our environments substantially reducing our ability to properly navigate our environments. In the LGN, without the care astrocytes and microglia provide to precisely sculpt and segregate visual connections, visual information entering our brains would likely not be faithfully transmitted to the cortex to be consciously represented leaving us experientially blind at worst or at the very least significantly impaired in terms of correctly identifying salient visual information in our environments, putting us at significant risk for injury. Without functional astrocytes in the visual cortex the connections from the thalamus relaying visual information would not be able to anchor into the correct locations in V1 leading to either conscious blindness or a highly disorganized representation of objects in the world. Without astrocytes and microglia to mediate plasticity and gate critical periods in the visual cortex, visual experience may never or may continually influence the wiring of our brains, leaving us with either impoverished and imprecise representations of our environments which lack the crucial contextual information that allows us to make appropriate perceptual decisions, or perpetually destabilizing our representations of the world and the binocular detection of important objects within it with important consequences for behavior.

In contrast to this, one can’t help but speculate as to what the future holds in terms of our ability to capitalize on the targeted manipulation of glial function throughout the visual system once we fully decode the nuances of their activity. Will we be able to fully repair damaged retinas, reversing or preventing blindness through modulating the function of Müller glia? Will we have the possibility of selectively enhancing and focusing our attention toward or away from specific stimuli in order to help accomplish specific goals or facilitate learning through the precise tuning of glial activity in the superior colliculus? Will it be possible to suppress or enhance specific types of visual information from reaching the visual cortex in order to help us selectively increase our ability to detect important visual cues in different environments by controlling the activity of glia in the thalamus? Will controlling glial activation in the visual cortex help us reopen critical periods of plasticity in a way that facilitates the functional reorganization necessary to recover from visual deficits in development or damage in adulthood or in ways that allow us to reformat our visual system on demand to function more optimally in a world continually populated by hyper-normal stimuli? What yet undiscovered possibilities remain to be found as we continue to develop an understanding of glial functions throughout the visual system? While we are only beginning to decode and understand the functional relevance of glial activity, it is eminently clear that our visual experience of the world is critically dependent on their function and that as research progresses, we are likely to continue to find novel important roles for glia throughout the visual system.

## Author Contributions

NB wrote the manuscript. DF and ER edited the manuscript. NB and DF created the figures. All authors contributed to the article and approved the submitted version.

## Conflict of Interest

The authors declare that the research was conducted in the absence of any commercial or financial relationships that could be construed as a potential conflict of interest.

## Publisher’s Note

All claims expressed in this article are solely those of the authors and do not necessarily represent those of their affiliated organizations, or those of the publisher, the editors and the reviewers. Any product that may be evaluated in this article, or claim that may be made by its manufacturer, is not guaranteed or endorsed by the publisher.
